# Acoustic Emission Detection and Analysis Method for Health Status of Lithium Ion Batteries

**DOI:** 10.3390/s21030712

**Published:** 2021-01-21

**Authors:** Kai Zhang, Jianxiang Yin, Yunze He

**Affiliations:** 1 Automotive and Transportation Engineering, Shenzhen Polytechnic, Shenzhen 518055, China; zhangkai@szpt.edu.cn; 2 College of Electrical and Information Engineering, Hunan University, Changsha 410082, China; pk4112413132@163.com; 3 State Grid Changde Power Supply Company, Changde 415000, China

**Keywords:** lithium ion battery, State of Health, acoustic emission, stress wave

## Abstract

The health detection of lithium ion batteries plays an important role in improving the safety and reliability of lithium ion batteries. When lithium ion batteries are in operation, the generation of bubbles, the expansion of electrodes, and the formation of electrode cracks will produce stress waves, which can be collected and analyzed by acoustic emission technology. By building an acoustic emission measurement platform of lithium ion batteries and setting up a cycle experiment of lithium ion batteries, the stress wave signals of lithium ion batteries were analyzed, and two kinds of stress wave signals which could characterize the health of lithium ion batteries were obtained: a continuous acoustic emission signal and a pulse type acoustic emission signal. The experimental results showed that during the discharge process, the amplitude of the continuous acoustic emission signal decreased with the increase of the cycle times of batteries, which could be used to characterize performance degradation; there were more pulse type acoustic emission signals in the first cycle of batteries, less in the small number of cycles, and slowly increased in the large number of cycles, which was in line with the bathtub curve and could be used for aging monitoring. The research on the health of lithium ion batteries by acoustic emission technology provides a new idea and method for detecting the health lithium ion batteries.

## 1. Introduction

**Figure 1:**
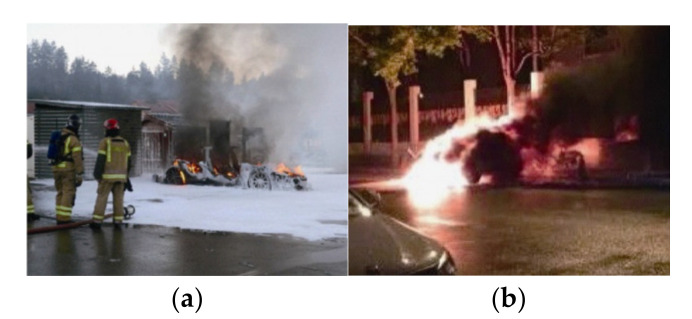
Lithium ion battery failure accident of electric vehicle [1]. (**a**) Battery catches fire when charging; (**b**) Self-ignition of the battery.

In recent years, energy and environmental problems have become increasingly prominent. The new energy vehicle industry and electronics industry put forward higher requirements for the performance of batteries. Lithium ion batteries are widely used in various industries due to their low environmental pollution, high specific energy, high cell voltage, and other good properties. Lithium ion batteries have been integrated into our lives, but battery failure has also caused a variety of safety accidents of electric vehicles, such as in Figure 1a, where a battery cought fire when charging, and in Figure 1b, which demonstrates the self-ignition of a battery [1]. Lithium ion battery failure will lead to very serious consequences, and at the same time, reduce people’s trust in lithium ion battery products. Usually, before the lithium ion battery accident occurs, a warning of battery failure state can be issued in advance by detecting the changes of internal parameters of the battery, and the faulty battery can be replaced in time to prevent the accident. In order to realize the early warning of the changes of the internal parameters of batteries, it is necessary to monitor the battery online. Therefore, it is necessary to find a safe, efficient, and economic method for detecting the health of lithium ion batteries online, provide a warning of the failure state of lithium ion batteries in advance, and reduce the occurrence of safety accidents of lithium ion batteries.

At present, the detection methods [2] for lithium ion batteries mainly include experimental methods [3], model methods [4,5], data-driven methods [6], and fusion methods [7]. Experimental methods can be classified into two categories: destructive testing and non-destructive testing. The destructive experimental method will destroy the structure of lithium ion batteries and make them unable to be used later; the non-destructive experimental method requires high precision of test data and high requirements of test environment [8,9]; the model method is used to establish the equivalent model of lithium ion batteries, simulate the charge-discharge process of batteries through various algorithms and the interaction between materials [10,11]. It is embedded in the battery management system to realize the online diagnosis function of battery SOH (State of Health), and thus has a high application prospect [12,13,14,15,16,17,18]; the data-driven method is based on the voltage, current, temperature, SOC (State of Charge), capacity, impedance and other data of lithium ion batteries during operation, realizes the determination of the health of lithium ion batteries in combination with algorithms, which is an important trend to realize battery state estimation and optimal management in the future [19,20]; the core idea of the fusion method is to combine, correlate and fuse multiple types of data, models or algorithms, and give full attention to their respective advantages to achieve a more precise and reliable collaborative estimation of lithium ion battery SOH. The implementation process of the fusion method is simple and fast, which shows good application prospects [21,22].

Among them, non-destructive testing is an excellent method to evaluate the internal or external physical and mechanical properties, various defects, and other technical parameters of an object. At present, the common measurement methods include X-ray diffraction (XRD), scanning electron microscope (SEM), transmission electron microscope (TEM), tomography, eddy current testing (ECT), magnetic particle testing (MT), ultrasonic testing (UT), acoustic emission testing (AET), and infrared thermal imaging (IRT), which are widely used in the industry.

In recent years, many experts and scholars have carried out experiments on lithium ion batteries by acoustic emission monitoring technology and obtained some research results [23,24,25]. Lemarié et al. studied the effect of an electrode binder and a current collector on battery performance [26]. In the experiment, the binders, namely polyvinylidene difluoride (PVdF) and carboxymethyl cellulose (CMC), and the collecting materials, namely carbon paper (CP) and aluminum foil, were combined pairwise to form four different kinds of electrode materials. The acoustic emission signals in the charge-discharge process of these materials were detected by acoustic emission, and the relationship between the number of acoustic emission signals and the number of cycles was analyzed to determine the performance of electrode materials. It was found in the experiment that the electrode with the combination of CMC and carbon paper had a more stable electrode performance. A. Tranchot et al. found three types of acoustic emission signals based on the acoustic emission characteristics of lithium ion batteries [27]: A series of short waves with high peak frequency (700 kHz) produced by the formation of microcracks on the surface of silicon particles; the signals generated by crack propagation into the composite membrane (400 kHz); the signals generated by increased electrode cracking (200 kHz) when c-Li_15_Si_4_ was formed. Fukushima et al. carried out an acoustic emission acquisition experiment on LiCoO_2_ batteries [28] and found that the resonant frequencies of acoustic emission signals were 110 kHz and 160 kHz, and the number of acoustic emission signals increased sharply when charging for 90 h, this had more to do with the fact that there were probably more and more side reactions occurring. Fukushima et al. studied the acoustic emission characteristics of lithium ion batteries with tin as an anode [29] and found low-frequency (50–100 kHz) acoustic emission signals generated by gas and high-frequency (100–300 kHz) acoustic emission signals generated by electrode cracking. Didier-Laurent et al. found that the frequency of acoustic emission signals generated by bubbles was between 100–200 kHz through the acoustic emission experiment of Ni-MN batteries [30], and this experimental result was also confirmed in an lithium ion battery experiment [31,32]. It should also be considered that the low-frequency range may also be due to other processes such as SEI (Solid Electrolyte Interface) formation, decomposition reactions, etc. [25,33,34].

In this paper, the generation mechanism of acoustic emission signals during the cycling of lithium ion batteries was elaborated based on the internal microstructure and electrochemical characteristics of lithium ion batteries with the building of a charge-discharge cycle platform and an acoustic emission detection platform of lithium ion batteries. In the experiment, the stress wave signals generated during the charge-discharge of lithium ion batteries were collected, and two kinds of acoustic emission signals could be used characterize the health of lithium ion batteries, namely a continuous acoustic emission signal and a pulse type acoustic emission signal. With the increase of battery cycle times, the amplitude of the former signal decreases gradually, and the latter signal decreases first and then increases. This phenomenon can be used to characterize the degradation degree of batteries. We believe that this study provides a novel idea and method for the health status detection of lithium ion batteries.

## 2. Generation Mechanism of Acoustic Emission Signal of Lithium ion Batteries

During the charge-discharge of lithium ion batteries, the internal structure of the battery changes due to the internal electrochemical reaction. Stress waves are produced by the rapidly released energy from local sources in the material. This kind of stress wave signal can be collected by acoustic emission equipment and converted into an acoustic emission signal for analysis.

### 2.1. Generation of Continuous Acoustic Emission Signal

During the charge-discharge of lithium ion batteries, the electrochemical reaction in the battery and the gas it generates will produce stress waves. This kind of stress wave signal exists in the whole process of a battery cycle. The waveform of the signal is regular and sinusoidal, which is known as a continuous acoustic emission signal.

The generation of the continuous acoustic emission signal may be related to electrode expansion, electrochemical reaction, and gas generation in an electrochemical reaction. Taking a ICR18650-22P lithium ion battery as an example, the positive electrode material of the battery is LiCoO_2_, and the negative electrode material is C. When the battery is charged, the equations of the reaction for positive and negative electrodes are shown in Equations (1) and (2), and the equations of discharge reaction are the inverse reaction of charge:

(1) $$\mathrm{Positive}\;\mathrm{electrode}:\;{\;\mathrm{Li}}_{1-x}{\mathrm{CoO}}_{2}+{\mathrm{xLi}}^{+}+{\mathrm{xe}}^{-}\to {\mathrm{LiCoO}}_{2}$$

(2) $$\mathrm{Negative}\;\mathrm{electrode}:\;{\mathrm{Li}}_{x}C_{6}\to 6C+{\mathrm{xLi}}^{+}+xe^{-}$$

In the research of lithium cobalt oxide ion battery, gas will be generated when the battery is overcharged. There are two reasons for gas generation [35,36]: one is the oxidative decomposition of electrolyte; the other is the dissolution of cobalt. By studying cobalt dissolution, it was found that the oxygen of CoO_2_ in electrolyte shows the structure of O3. When the battery is overcharged, the structure of oxygen is transformed from O3 into O1. CoO_2_ with a structure of O1 is unstable thermodynamically and easy to decompose, which leads to the generation of oxygen. The equation of chemical reaction for the transformation of the structure of LiCoO_2_ electrode from O3 to O1 is shown in Equation (3) [37]:

(3) $${2\mathrm{Li}}_{x}{\mathrm{CoO}}_{2}(O3)\;+{4\mathrm{xH}}^{+}\to \left(2-x){\mathrm{CoO}}_{2}(O1\right)+{\;\mathrm{xCo}}^{2+}+{\;2\mathrm{xLi}}^{+}\;+{2\mathrm{xH}}_{2}O$$

In Equation (3), CoO_2_ (O1 phase) forms on the surface of the electrode decomposes into cobalt oxide and releases oxygen, in which cobalt shows a low oxidation state (+2/+3). The reaction is shown in Equation (4) below [38]:

(4) $${3\mathrm{CoO}}_{2}(O1)\;\to {\mathrm{Co}}_{3}O_{4}\;+{\;O}_{2}$$

When there is moisture in the battery, the moisture will consume lithium ions, which will also generate gas. The equation of the chemical reaction is shown in Equation (5):

(5) $${\mathrm{LiCoO}}_{2}+C+H_{2}O\to {\mathrm{LiC}}_{6}+{\mathrm{LixCo}}_{2}+\mathrm{LiOH}+H_{2}$$

### 2.2. Generation of Pulse Type Acoustic Emission Signal

**Figure 2:**
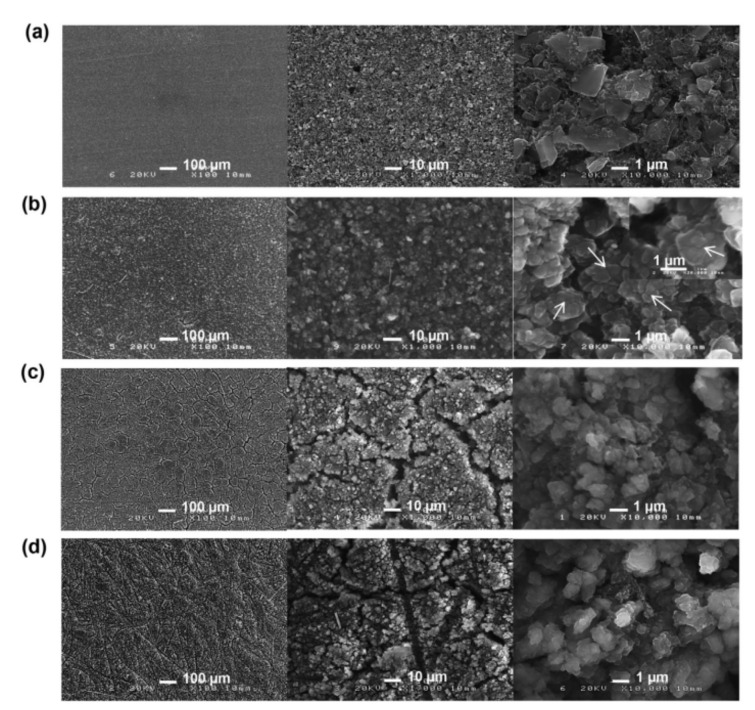
Electrode change [27]: (**a**) Not cycled; (**b**) The first discharge; (**c**) The first charge; (**d**) 10 cycles.

Pulse type acoustic emission signals are a kind of rapidly decaying signal. In lithium ion batteries, it is mainly produced by the formation of electrode cracks. Taking a silicon-based lithium ion battery as an example, a micrograph of the surface of a silicon negative electrode before and after the cycle of silicon-based lithium ion battery is shown in Figure 2 [27]. Figure 2a shows the silicon-based surface before the cycle of a lithium ion battery, and Figure 2b–d shows the silicon-based surface after the first discharge, the first charge, and 10 cycles, respectively. It can be seen from the micrograph that the surface of the electrode of the lithium ion battery cracks and age continuously during cycling.

Electrode cracking generates a stress wave, as described in reference [29], which is the pulse acoustic emission signal generated by the formation of SnO electrode crack.

## 3. Experimental System and Experimental Design

**Figure 3:**
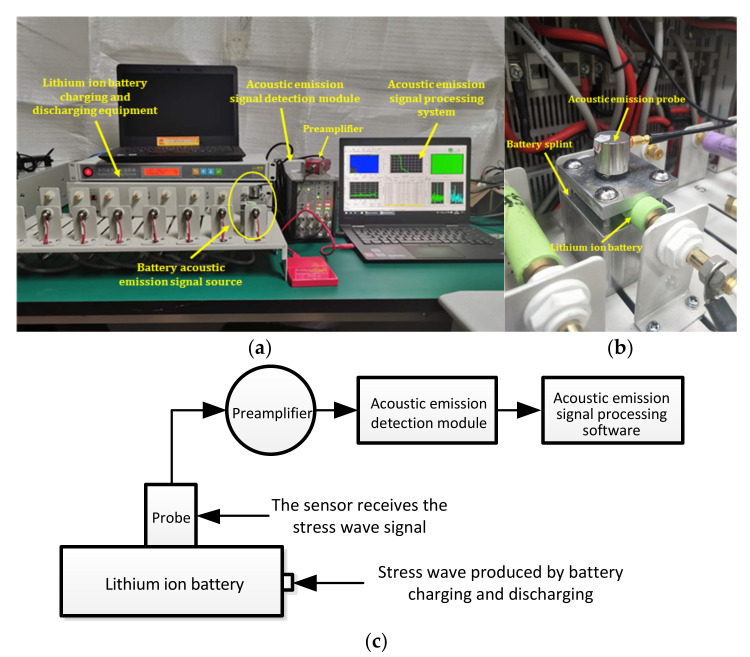
Test system diagram. (**a**) Experimental device diagram; (**b**) Position relationship between the clamp plate and the lithium ion battery; (**c**) Block diagram to the experimental.

The experimental system mainly consisted of a charge-discharge module for lithium ion batteries and a measurement platform for acoustic emission. Figure 3 is the system diagram of the acoustic emission detection experiment for lithium ion batteries. The battery charge-discharge module included a Panasonic ICR18650-22P lithium ion battery, aluminum battery fixture, Neware battery tester (CT-4008-5V20A-A), etc. The acoustic emission measurement platform included an ASMY-6 measuring instrument (bandwidth: 10 MHz) produced by Vallen in Germany, a VS-45H piezoelectric sensor (bandwidth: 20 kHz–450 kHz), an AEP5 preamplifier (34 dB), and a notebook computer equipped with adaptive acoustic emission acquisition system software.

The purpose of each part of the acoustic emission measurement platform is described in Figure 3. As shown in Figure 3b, the aluminum fixture was set between the lithium ion battery and the acoustic emission sensor, and the contact surface between the aluminum fixture and the battery was designed to be the same as the arc of the battery, which was convenient for the aluminum fixture to contact with the battery more fully. The reason for selecting this fixture is that the metal has better acoustic conductivity. A layer of coupling agent is applied between the aluminum fixture and the sensor and between the aluminum fixture and the battery to reduce the loss of acoustic signal on the contact surface. The sensor was used to receive the acoustic signal collected from the surface of the aluminum fixture and convert the acoustic signal into an electrical signal. The preamplifier was the accessory of the acoustic emission instrument, which was mainly used to amplify the electrical signal from the sensor. The main function of the acoustic emission instrument was to analyze the detected electrical signal and convert the signal into a signal that can be recognized by the processing module software. The signal processing module was mainly used to observe and analyze the acoustic emission signal.

In the experiment, lithium ion batteries were divided into two groups, A and B, with different cycle conditions in each group. The cycle charge-discharge mode was constant current charge-constant voltage charge-constant current discharge, and the experimental charge-discharge mode was constant current charge first and then constant voltage charge. The cycle mode of fast charge and fast discharge was adopted in Group A, and rated charge-discharge parameters were used in Group B for cycling. The cycle parameters are shown in Table 1. The cycle charge-discharge current is the cycle parameter of a lithium ion battery in a non-acquisition cycle, and the experimental charge-discharge current is the cycle parameter of a lithium ion battery in the acquisition cycle of an acoustic emission signal. The acquisition cycle of the acoustic emission signal of lithium ion batteries is shown in Table 2.

Each lithium ion battery was fixed with an aluminum fixture, and the acoustic emission VS-45H piezoelectric sensor was attached to the surface of the aluminum fixture with a coupling agent. The acoustic emission signal detection experiment was carried out in the acquisition cycle of the battery plan shown in Table 2, and real-time monitoring was carried out in the acoustic emission signal processing software of the notebook. The acquired acoustic emission signal data was saved and processed by the signal processing software, Matlab.

## 4. Results

In order to reduce the influence of environmental noises on acoustic emission signals, it was necessary to adopt certain technical means to deal with noise. The specific methods were as follows: When the charge-discharge experiment of a battery had not been carried out, the sensor was used to acquire the ambient noise, and the signals generated by the ambient noise were transmitted to the signal processing module for analysis. It was found that the noise signals were below 30 dB. In the experiment, the acoustic emission signals were generally above 32 dB, and the threshold could be set to 32 dB to reduce the effect of noise signals on the experimental signals. In the time-frequency analysis of noise signals, it was found that the amplitude of noise signals was far less than the amplitude of experimental signals, so it could be considered that the noise signals had no effect on the experimental signals. In addition, in order to ensure that the amplitude of the acoustic emission (AE) signal was much larger than that of the noise signal, the probe was placed in the middle of the battery with the strongest AE signal in previous experiments.

### 4.1. Component Analysis of Acoustic Emission Signal

According to the characteristics of the time domain and frequency domain of the signals, the acoustic emission signal can be classified into a continuous acoustic emission signal and a pulse type acoustic emission signal.

**Figure 4:**
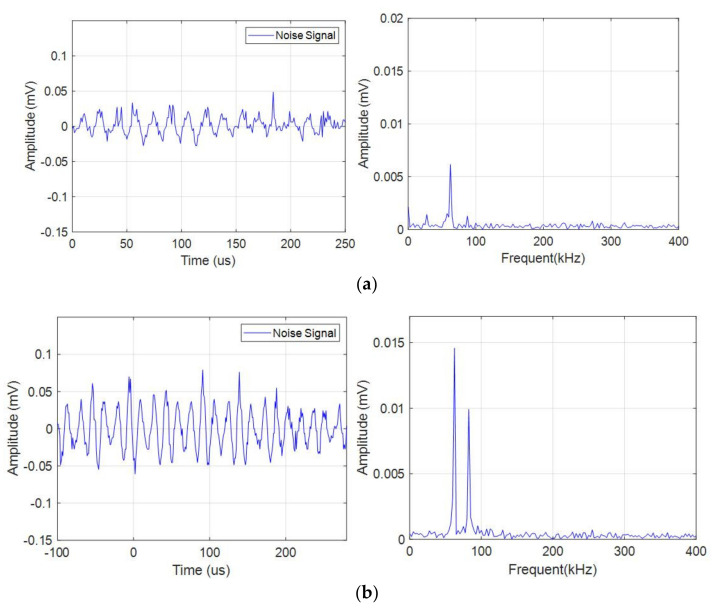
Time domain-frequency domain diagram of continuous acoustic emission signal. (**a**) Charge process; (**b**) Discharge process.

The continuous acoustic emission signals had a large quantity and low amplitude (compared with the pulse type acoustic emission signal), which existed in the whole battery cycle. At the same time, the characteristics of acoustic emission signal during charge and discharge were different, as shown in Figure 4a,b. During discharge, the amplitude of the acoustic emission signal was high, and there were two dominant frequencies of 60 kHz and 88 kHz, while during charge, the amplitude of the acoustic emission signal was low, and there was only one dominant frequency of 60 kHz. The acoustic emission signal with a dominant frequency of 60 kHz in both charge and discharge might be related to electrode expansion, and the dominant frequency of 88 kHz only appeared in discharge might be caused by different gases produced by electrochemical reaction during discharge.

**Figure 5:**
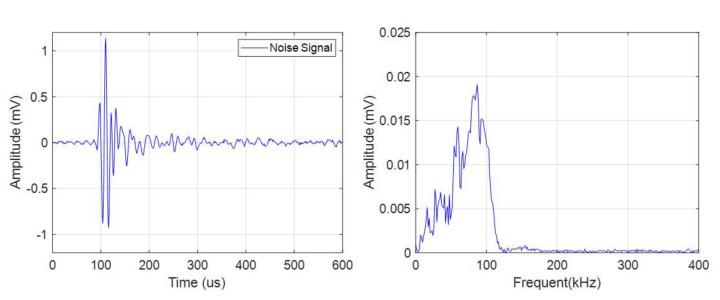
Time domain-frequency domain diagram of pulse type acoustic emission signal.

The pulse type acoustic emission signals had a small quantity, high amplitude, and wide frequency band. There were more pulse type acoustic emission signals during charge than discharge. As shown in Figure 5, the frequency band of the signal was in the range of 0–150 kHz.

### 4.2. Analysis of Continuous Acoustic Emission Signal

Since the characteristics of continuous acoustic emission signals during charge and discharge were different, it was necessary to analyze the acoustic emission signals during charge and discharge separately when exploring the relationship between continuous acoustic emission signals and the health of a battery.

**Figure 6:**
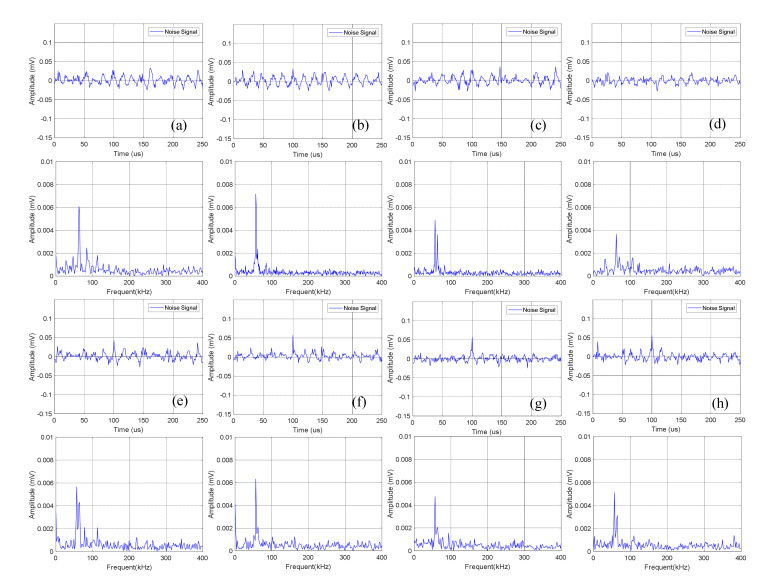
Time domain-frequency domain diagram of 0–600 cycles of charge of Group A batteries: (**a**) 0 cycles; (**b**) 10 cycles; (**c**) 100 cycles; (**d**) 200 cycles; (**e**) 300 cycles; (**f**) 400 cycles; (**g**) 500 cycles; (**h**) 600 cycles.

By analyzing Figure 6a,b, the amplitude signals in the time domain program and frequency domain program could be extracted. Since it was easy to extract the amplitude information in the frequency domain diagram (it was only necessary to extract the amplitude signal of the fixed dominant frequency), and the amplitude in both time domain and frequency domain could achieve the purpose of characterizing continuous acoustic emission signal, in this paper, statistics were only made on the amplitude of acoustic emission signal in the frequency domain, and this method was also used in the analysis of the amplitude of other continuous acoustic emission signals.

#### 4.2.1. Continuous Acoustic Emission Signal during Charge

The time domain-frequency domain diagram of acoustic emission signals during the 0–600 cycles of charge of Group A batteries is shown in Figure 6. It can be observed from the frequency domain diagram that the acoustic emission signals during charge were mainly composed of waveform with a frequency of 60 kHz.

Statistics of the amplitude of the acoustic emission signals of Group A during charge are shown in Table 3. It can be seen that the continuous acoustic emission signals generated during charge changed little with the increase of the number of battery cycles, and the dominant frequency amplitude was about 0.005 mV.

**Figure 7:**
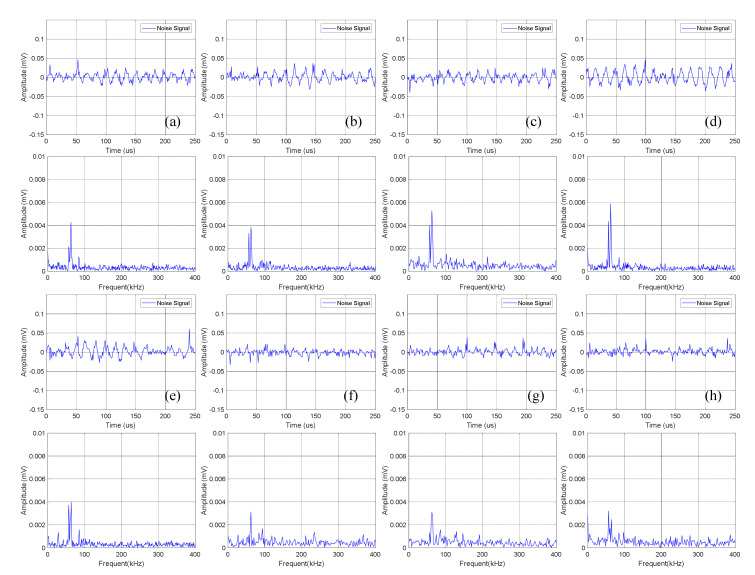
Time domain-frequency domain diagram of 0–600 cycles of charge of Group B batteries: (**a**) 0 cycles; (**b**) 10 cycles; (**c**) 100 cycles; (**d**) 200 cycles; (**e**) 300 cycles; (**f**) 400 cycles; (**g**) 500 cycles; (**h**) 600 cycles.

The time domain-frequency domain diagram of acoustic emission signal changes of Group B batteries during the 0–600 cycles of charge is shown in Figure 7.

Statistical analysis was performed on the amplitude of the dominant frequency of the frequency domain in Figure 7, as shown in Table 4. It can be seen that the amplitude data of Group B is slightly lower than that of Group A batteries, which is about 0.004 mV.

The amplitude of continuous acoustic emission signals of Group A and Group B during charge changed little with the increase of battery cycles, and the amplitude changes were not monotonic but discrete. Therefore, it was infeasible to determine the health of the lithium ion batteries by the amplitude information of the continuous acoustic emission signals during charge.

#### 4.2.2. Continuous Acoustic Emission Signal during Discharge

**Figure 8:**
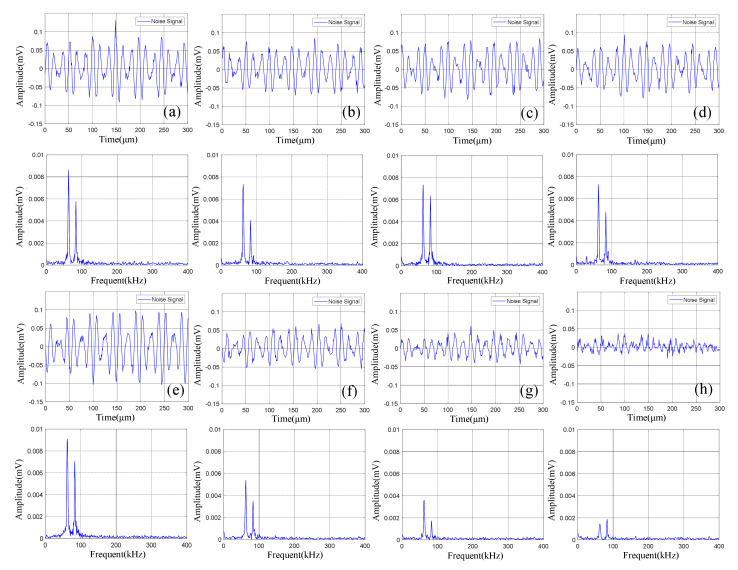
Time domain-frequency domain diagram of 0–600 cycles of discharge of Group A batteries: (**a**) 0 cycles; (**b**) 10 cycles; (**c**) 100 cycles; (**d**) 200 cycles; (**e**) 300 cycles; (**f**) 400 cycles; (**g**) 500 cycles; (**h**) 600 cycles.

The time domain-frequency domain diagram of acoustic emission signals during the 0–600 cycles of discharge of Group A batteries is shown in Figure 8.

**Figure 9:**
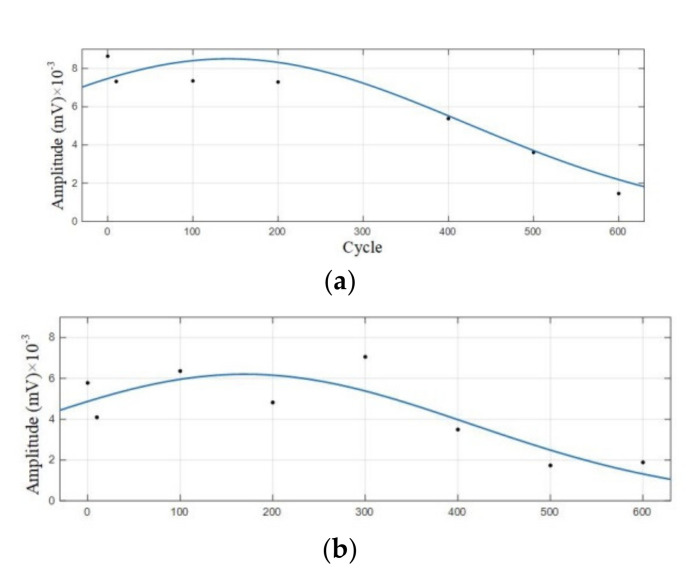
Amplitude variation curve of the 0–600 cycles of Group A batteries. (**a**) Amplitude variation curve with a dominant frequency of 60 kHz; (**b**) Amplitude variation curve with a dominant frequency of 88 kHz.

Statistical analysis was performed on the amplitude of the two dominant frequencies in the frequency domain diagram, as shown in Table 5. The curve fitting was carried out according to the amplitude information obtained to get the amplitude variation curve of the lithium ion batteries discharge, as shown in Figure 9.

It can be observed from Figure 8 that in the discharge state, the acoustic emission signals had two dominant frequencies, 60 kHz and 88 kHz, in which the amplitude of the dominant frequency of 60 kHz was greater than that of 88 kHz. It can be seen in Figure 9 that the amplitude of the two dominant frequencies showed a decreasing trend with the increase of cycle number, and the amplitude changed little at 0–300 cycles. After more than 400 cycles of battery, the amplitude of the two dominant frequencies began to decrease significantly.

**Figure 10:**
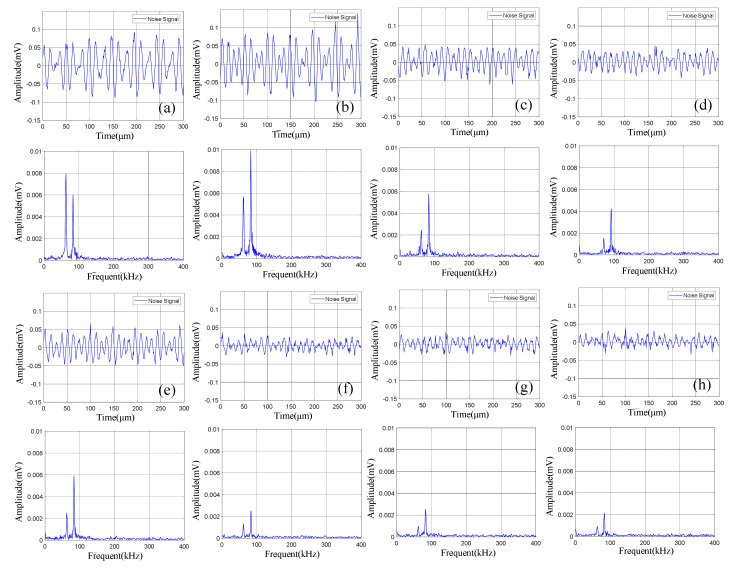
Time domain-frequency domain diagram of 0–600 cycles of discharge of Group B batteries: (**a**) 0 cycles; (**b**) 10 cycles; (**c**) 100 cycles; (**d**) 200 cycles; (**e**) 300 cycles; (**f**) 400 cycles; (**g**) 500 cycles; (**h**) 600 cycles.

The time domain-frequency domain diagram of acoustic emission signals during the 0–600 cycles of discharge of Group B batteries is shown in Figure 10.

The acoustic emission signals of Group B batteries during discharge also had two dominant frequencies of 60 kHz and 88 kHz. Compared with Group A batteries in the discharge state, the amplitude of the acoustic emission signals with a dominant frequency of 88 kHz of Group B batteries was higher than 60 kHz.

**Figure 11:**
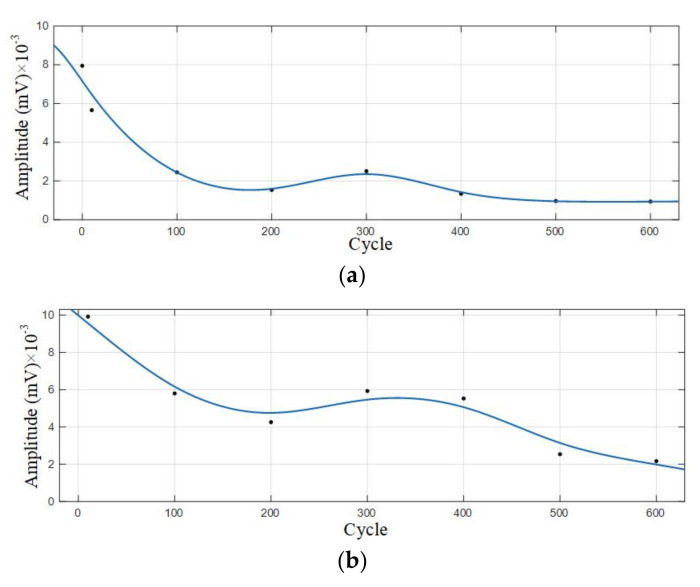
Amplitude variation curve of the 0–600 cycles of Group B batteries. (**a**) Amplitude variation curve with a dominant frequency of 60 kHz; (**b**) Amplitude variation curve with a dominant frequency of 88 kHz.

Statistical analysis was performed on the amplitude of the two dominant frequencies in the frequency domain diagram, as shown in Table 6. The curve fitting is carried out on amplitude data to get the amplitude variation curve, as shown in Figure 11.

According to the analysis of amplitude variation curve, the amplitude of Group B signals also showed a trend of gradual decrease with the increase of cycle number, and the amplitude with a dominant frequency of 88 kHz decreased most significantly in the 400–500 cycles; the amplitude with a dominant frequency of 60 kHz decreased significantly in the 10–100 cycles, and the amplitude decreased slowly in the 100–600 cycles.

By analyzing the time-frequency diagram of the acoustic emission signals of Group A and B batteries during discharge, it can be found that the acoustic emission signals during discharge had the following characteristics: (1) The signals had two dominant frequencies: 60 kHz and 88 kHz; (2) Compared with the acoustic emission signals during charge, the amplitude of the acoustic emission signals during discharge was higher; (3) The amplitude of the signals decreased with the increase of cycle number. By comparing the acoustic emission signals of lithium ion batteries of Group A and B in different cycle states, it could be observed that: (1) The amplitude of acoustic emission signals with a dominant frequency of 88 kHz was lower than that of 60 kHz during discharge for batteries with cycle parameters of fast charge and fast discharge (Group A); the amplitude of acoustic emission signals with a dominant frequency of 88 kHz was higher than 60 kHz during discharge for batteries with cycle parameters of rated charge-discharge (Group B); (2) The amplitude of acoustic emission signals of Group A batteries decayed rapidly in the 300–400 cycles, while the decaying range of Group B batteries appeared in the range of 400–500 kHz, which indicates that the decaying of Group A batteries was faster in the cycles of fast charge and fast discharge.

The activity of the internal electrochemical reaction of lithium ion batteries during charge and discharge can reflect the health of a lithium ion battery. Acoustic emission technology can acquire the stress wave signals generated by electrochemical reaction. In the experiment, it was found that the amplitude of continuous acoustic emission signals decreased with the number of battery cycles, which was consistent with the characteristics of the fading of internal electrochemical reaction when the lithium ion battery was gradually aging and could be used to monitor the performance degradation.

### 4.3. Analysis of Pulse Type Acoustic Emission Signal

The pulse type acoustic emission signal is generated when cracks in a battery are formed, which can be detected during both charge and discharge of a battery. In the analysis of the acoustic emission signal, the amplitude of the pulse type acoustic emission signal was irregular, and it was impossible to form a corresponding relationship between amplitude information and cycle number. Therefore, the amplitude analysis method used in continuous acoustic emission signal was no longer suitable for studying the relationship between the pulse type acoustic emission signal and the health of a lithium ion battery. Based on the principle of electrode crack formation and crack evolution, the health of a lithium ion battery was studied by measuring the number of pulse type acoustic emission signals.

**Figure 12:**
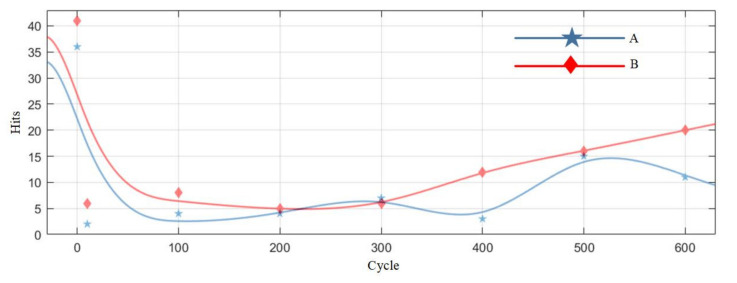
The variation curve of the number of pulse type acoustic emission signals.

**Figure 13:**
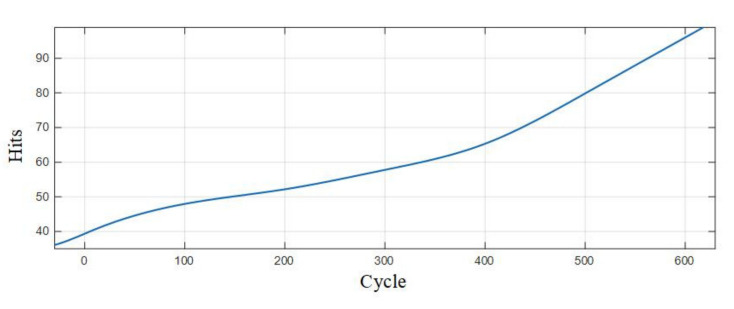
Cumulative curve of the number of pulse type acoustic emission signals.

Table 7 is the statistics of the number of pulse type acoustic emission signals detected in the 0–600 cycles of Group A and B batteries. Figure 12 is the fitted curve of the variation of the pulse type acoustic emission signal with cycle number, which directly shows the change in the number of pulse type acoustic emission signals of lithium ion batteries in different health states. Figure 13 is the cumulative pulse type acoustic emission signal curve fitted according to the experimental data. It can be seen from the figure that the increasing speed of pulse type acoustic emission signal is fast-slow-fast.

By analyzing the curves, it could be found that the number of pulse type acoustic emission signals was the largest during the first charge-discharge of lithium ion batteries, and the acoustic emission signals decreased sharply in the cycle after the first charge-discharge; in the 10–300 cycles, the number of pulse type acoustic emission signals was very small, which was in a relatively stable stage; after the lithium ion battery cycled for more than 300 times, the number of pulse type acoustic emission signals showed a slow-growth trend.

The analysis was then viewed from the perspective of the battery structure change. During the first cycle of a lithium ion battery, a large number of cracks were generated with the intercalation of lithium ions into the electrode, which resulted in a large number of pulse type acoustic emission signals; at the same time, SEI can transport lithium ions into the graphite surface, which is a kind of protective layer that can penetrate lithium ions. Lithium is consumed in the early stage of SEI formation, which has a certain impact on the capacity of the battery. SEI is also an active acoustic process, but the specific acoustic emission law needs to be further studied.

Viewing the analysis from the perspective of a mathematical model, by observing the fitted curve in Figure 12, it could be found that the variation of the number of pulse type acoustic emission signals with time presented with a concave curve, higher at both ends and lower in the middle, which is similar to a bathtub curve. The bathtub curve refers to the curve of product failure rate changing with time in the whole life cycle from putting into operation to scrapping. According to the model description of a bathtub curve, the failure rate of a product can be divided into three stages, among which the failure rates in the early failure period and loss failure period are the highest, and the failure rate in the accidental failure period is lower. The three stages of the curve of the number of the pulse type acoustic emission signals fitted from the pulse type acoustic emission signals detected in the experiment are one-to-one corresponding to those of the bathtub curve. In the early stage of the cycle, there were more pulse type acoustic emission signals and a higher failure rate of batteries; in the middle of the cycle, there were fewer such signals, and the battery operation was stable; in the aging stage of battery, the number of such signals increased gradually again, and the performance of battery decreased sharply.

The variation curve of pulse type acoustic emission signal accords with the trend of product failure and the change rule of the battery electrode structure, which could be used as a method to determine the health of lithium ion batteries.

## 5. Conclusions

Through the analysis of theoretical and experimental results, the following conclusions can be drawn:

(1) Continuous acoustic emission signals exist in the whole charge-discharge process of batteries, and the signal during charge was different from that during discharge. The dominant frequency of charge could only be 60 kHz, while that of discharge was 60 kHz and 88 kHz. The dominant frequency of 60 kHz appeared in the whole charge-discharge process, which may be related to electrode expansion; 88 kHz only appeared during discharge, which may be related to the electrochemical reaction during discharge. By analyzing the amplitude of the two kinds of signals, it was found that there was no obvious correlation between the amplitude of acoustic emission signal during charge and the number of battery cycles, while the amplitude of discharge gradually decreases with the increase of cycle number. Therefore, the amplitude information of continuous acoustic emission can be used as a method for detecting the performance degradation of lithium ion batteries.
(2) The continuous acoustic emission signals detected during discharge of batteries with different cycle states (with different internal structures of batteries) are different: The amplitude with a dominant frequency of 88 kHz is lower than that of 60 kHz for batteries with the cycle parameters of fast charge and fast discharge; the amplitude with a dominant frequency of 88 kHz was higher than 60 kHz for batteries with the cycle parameters of rated charge-discharge; therefore, it is necessary to consider the historic cycle parameters of battery when detecting the health of lithium ion batteries through the amplitude of continuous acoustic emission signals.
(3) The pulse type acoustic emission signal mainly appeared in the first charge-discharge process of batteries. It had a small and stable number when the cycle number was small and increased gradually when the cycle number was large, which has a certain corresponding relationship with the number of battery cycles and is in line with the bathtub curve model. Therefore, pulse type acoustic emission signal could also be used as a method for detecting the aging state of lithium ion batteries.

It should be pointed out that, limited to the authors’ research conditions, the combination of acoustic emission data and electrochemical data was not considered in current experiments. More valuable results may be obtained if electrochemical data are properly introduced in a follow-up study.

## 6. Patents

Based on the work in this paper, we have applied for and authorized a Chinese patent: an acoustic emission detection system for lithium ion battery health status (Patent No. CN201921466156.3).

## Figures and Tables

**Table 1:** Setting of cycle parameters.

Cycle Parameters	Cyclic Charge Current/C-Rate	Cyclic Charge Voltage	Cyclic Discharge Current/C-Rate	Test Charge Current/C-Rate	Test Discharge Current/C-Rate
Group A	2150 mA/1 C	4.2 V	4300 mA/2 C	2150 mA/1 C	4300 mA/2 C
Group B	1075 mA/0.5 C	4.2 V	2150 mA/1 C	2150 mA/1 C	4300 mA/2 C

**Table 2:** Signal acquisition plan.

	Cycle Number/Cycles of Lithium Ion Batteries							
Acoustic emission detection	0	10	100	200	300	400	500	600

**Table 3:** Amplitude information of acoustic emission signals of Group A batteries during charge.

Number of Cycles	0	10	100	200	300	400	500	600
Amplitude of dominant frequency in frequency domain/mV × 10^−3^	6.26	7.2	4.76	3.81	5.93	6.63	4.95	6.67

**Table 4:** Amplitude information of acoustic emission signals of Group B batteries during charge.

Cycle Number	0	10	100	200	300	400	500	600
Amplitude of dominant frequency in frequency domain/mV × 10^−3^	3.85	3.07	5.33	6.01	4.15	3.17	3.24	4.21

**Table 5:** Amplitude information of acoustic emission signals of Group A batteries during discharge.

Number of Cycles	0	10	100	200	300	400	500	600
Amplitude of dominant frequency of 60 kHz/mV × 10^−3^	8.64	7.32	7.35	7.29	9.11	5.38	3.61	1.46
Amplitude of dominant frequency of 88 kHz/mV × 10^−3^	5.78	4.09	6.36	4.82	7.06	3.49	1.73	1.88

**Table 6:** Amplitude information of acoustic emission signals of Group B batteries during discharge.

Number of Cycles	0	10	100	200	300	400	500	600
Amplitude of dominant frequency of 60 kHz/mV × 10^−3^	7.95	5.66	2.45	1.54	2.51	1.34	0.97	0.94
Amplitude of dominant frequency of 88 kHz/mV × 10^−3^	6.03	9.92	5.8	4.26	5.93	2.53	2.54	2.17

**Table 7:** Statistics of the number of pulse acoustic emission signals.

Number of Cycles	0	10	100	200	300	400	500	600
Group A	36	2	4	4	7	3	15	11
Group B	41	6	8	5	6	12	16	20

## Data Availability

Not applicable.
